# Immunological effects of glutamine supplementation in polytrauma patients in intensive care unit

**DOI:** 10.1186/s44158-022-00068-1

**Published:** 2022-09-24

**Authors:** Antonella Cotoia, Leonarda Pia Cantatore, Renata Beck, Livio Tullo, Donatella Fortarezza, Flavia Marchese, Giuseppe Ferrara, Gilda Cinnella

**Affiliations:** Department of Medical and Surgical Sciences, Anesthesia and Intensive Care Unit, Policlinico Riuniti Foggia, University of Foggia, Foggia, Italy

**Keywords:** Glutamine, Polytrauma, Immunoglobulin A, CD3+/CD4+ T helper lymphocytes, CD3+/CD8+ T suppressor lymphocytes, CD3+/CD19+ B lymphocytes

## Abstract

**Background:**

In polytrauma intensive care unit (ICU) patients, glutamine (GLN) becomes a “conditionally essential” amino acid; its role has been extensively studied in numerous clinical trials but their results are inconclusive.

We evaluated the IgA-mediated humoral immunity after GLN supplementation in polytrauma ICU patients.

**Methods:**

All consecutive patients with polytrauma who required mechanical ventilation and enteral nutrition (EN) provided within 24 h since the admission in ICU at the University Hospital of Foggia from September 2016 to February 2017 were included.

Thereafter, two groups were identified: patients treated by conventional EN (25 kcal/kg/die) and patients who have received conventional EN enriched with 50 mg/kg/ideal body weight of alanyl-GLN 20% intravenously.

We analysed the plasmatic concentration of IgA, CD3+/CD4+ T helper lymphocytes, CD3+/CD8+ T suppressor lymphocytes, CD3+/CD19+ B lymphocytes, IL-4 and IL-2 at admission and at 4 and 8 days.

**Results:**

We identified 30 patients, with 15 subjects per group. IgA levels increased significantly in GLN vs the control group at T0, T4 and T8. CD3+/CD4+ T helper lymphocyte and CD3+/CD8+ T suppressor lymphocyte levels significantly increased in GLN vs the control group at T4 and T8. CD3+/CD19+ B lymphocyte levels increased significantly in GLN vs the control group only at T8. IL-2 and IL-4 levels showed no significant differences when comparing GLN with the control group.

**Conclusions:**

Our study showed that there was an improvement in humoral and cell-mediated immunity with GLN supplementation in polytrauma ICU patients using recommended doses.

## Background

Glutamine (GLN) is classified as a non-essential amino acid and it is released from skeletal muscle to be a constituent of proteins [1]. Furthermore, GLN acts as an immune stimulator as an essential component for lymphocyte proliferation and cytokine production, macrophage phagocytic and secretory activities, and neutrophil bacterial killing [2].

Under stable conditions, GLN can be produced in sufficient amounts and stored in the muscle tissue. These stores actually represent greater than 50% of the total free amino acid pool in the body.

However, GLN levels normally decrease in intensive care unit (ICU) patients both due to a hypercatabolic status, especially in trauma and sepsis, and an increased requirement by the gut, the immune system, liver and kidneys which exceeds the individual’s ability to produce it in sufficient amounts. In critically ill patients, GLN may even become a “conditionally essential” amino acid [3]: it is considered a “fuel for the immune system”, where a low blood concentration may impair immune cell function, resulting in poor clinical outcomes and increased risk of mortality, and so its supplementation is recommended in ICU patients [4]. Interestingly, some authors showed that GLN supplements of 10–20 g/day (or with a high dose >0.2g/kg/day) plus standard enteral nutrition (EN) formulas reduced the rates of pneumonia, sepsis and bacteraemia in ICU patients with shortened hospital stays, better immune function and lower hospital costs [5–12]. It is important to note that humoral immunity is a process of adaptive immunity mediated by immunoglobulins such as IgA secreted by B lymphocytes [5]. Cell-mediated immunity is responsible for destroying the intracellular pathogens with T lymphocytes, which consequently produce inflammatory mediators such as interleukin. More clinical trial and new studies on these topics are necessary to focus on cell-mediated and humoral immune responses in severely injured trauma patients and rule on GLN depletion on immune functioning [9–14].

We hypothesize that GLN could enhance both humoral and cell-mediated immunity. The aim of our study is to investigate the effect of GLN-enriched EN on the cell-mediated and humoral immune system in ICU polytrauma patients.

## Methods

Ethical approval was obtained from the Hospital Ethical Committee at the University Hospital of Foggia, on September 12, 2019 (83/C.E./2019), and written informed consent was obtained from the patient during a planned follow-up visit.

The study included all consecutive patients with polytrauma (Injury Severity Score > 15) who required mechanical ventilation and EN provided within 24 h since the admission in ICU at the University Hospital of Foggia from September 2016 to February 2017.

Exclusion criteria were age <18 years, renal failure (creatinine > 180 μmol/L) or liver failure (bilirubin > 40 mol/L, alanine aminotransferase > 100 units/L and gamma glutamyl transferase > 100 units/L), patients who underwent major abdominal surgery or receiving systemic steroids, known history of immunologic disorders pregnancy and patient receiving supplemental parenteral nutrition.

Thereafter, two groups were identified: the control group received conventional EN. The caloric target of 25 kcal/kg/die with a protein target of 1.2 g/kg/die was progressively reached in the first week of ICU stay. The GLN group was treated with conventional EN enriched with 50 mg/kg/ideal body weight (IBW) of alanyl-GLN 20% (Dipeptiven, Fresenius-Kabi, Bad Homburg, Germany) intravenously in a central venous line (GLN group). GLN infusion was administrated on 7 consecutive days [6].

The IBW was calculated using the Broca formula [7]: for men, IBW= [height (cm)−100] − [height (cm)−100] × 10%; for women, IBW= [height (cm)−100] + [height (cm)−100] × 15%.

Demographics, Simplified Acute Physiology Score (SAPS) II, Acute Physiologic Assessment and Chronic Health Evaluation Score (APACHES) II and Injury Severity Score (ISS), ICU length of stay and mortality were retrospectively recorded.

Additionally, albumin, total plasmatic proteins and plasmatic urea levels were recorded at admission in ICU. The plasmatic concentration of IgA, CD3+/CD4+ T helper lymphocytes, CD3+/CD8+ T suppressor lymphocytes, CD3+/CD19+ B lymphocytes, IL-4 and IL-2 were analysed at T0 (T0), at 4 (T4), and at 8 (T8) days after ICU admission.

### Statistical analysis

We identified a sample size of 30 patients, with 15 subjects per group.

The normality of distribution was assessed by the *Shapiro-Wilkinson test*.

Since we found all of the data normally distributed, the data were expressed as means ± SD. A paired sample *t*-test was used to detect changes within the groups.

Data were analysed using one-way *ANOVA* and repeated measurement analysis of variance.

Differences between the groups at each time point were examined post hoc using an independent *sample t-test*.

A value of *p*<0.05 was considered statistically significant.

Statistical analysis was performed by *Statistical Package for the Social Sciences* (SPSS Inc., Chicago, IL) version15.0 for Windows.

## Results

Forty-six patients were screened for eligibility. Five out of forty-six patients were not eligible due to an ICU stay < 96 h and 11 were excluded because they died within 8 days in ICU.

Thirty patients were enrolled and divided into two groups based on different treatments.

No patients reported any intolerance, hypersensitivity or side effects related to treatment.

No difference was found in age, gender and weight of patients’ characteristics between the two groups.

No significant differences were found either in ISS, SAPS or APACHE II score.

The ICU length of stay and 28-day mortality were similar in both groups.

Furthermore, there was no significant difference in mechanical ventilation (MV) time between GLN and control groups (Table 1).

**Table 1:** Demographic, laboratory and clinical characteristics

	Control EN Group 1 n.15	GLN-enriched EN Group 2 n.15	*p* value
Age (y)	53.5±11.4	54.0±11.7	0.57
Weight (kg)	76±16.4	74.53±16.9	0.48
Height (cm)	165±9.2	165±7.5	0.22
BMI (kg/cm^2^)	28.7±8.1	29.1±8.9	0.19
Sex (M/F)	8/7	10/5	0,17
Albumin (g/dl)	2.5±0.9	2.8±0.6	0.82
Proteins (g/dl)	5.3±0,9	6.2±0.8	0.91
Plasmatic urea level (mmol/litre)	41±9.2	45±8.1	0.46
GCS score	11±2	12±3	0.40
ISS score	27.5±10.1	40.9±19.8	0.09
SAPS score	45.4±12.1	45.8±9.8	0.9
APACHES II score	23.6±3.6	21.4±6.3	0.76
ICU length of stay (days)	25±5	20±7	0.39
ICU mortality, *n* (%)	6 (40)	5 (33.3)	0.4
MV time (d)	9.26±1.3	9±1.4	0.31

**Fig. 1:**
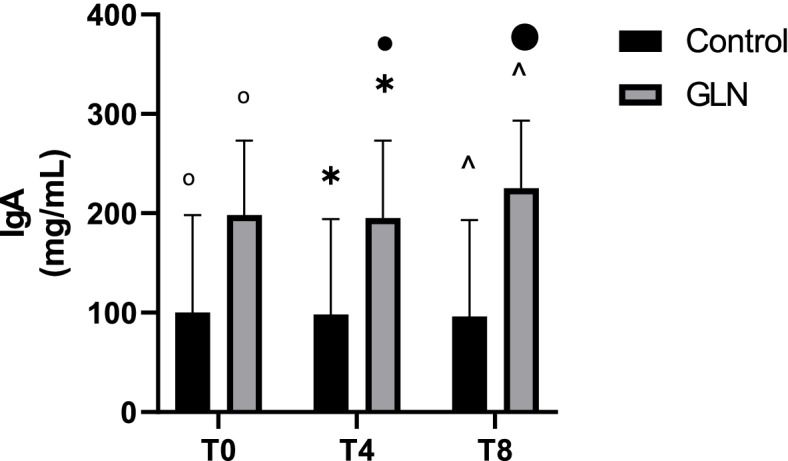
IgA levels in control and glutamine (GLN) groups. Data are presented as mean ± SD. °, * and  ^ indicate a significant difference in GLN vs control at T0 (*p* < 0.05), at T4 (*p* < 0.05), and at T8 (*p* < 0.01); • indicates a significant difference (*p* < 0.05) in the GLN group between T4 and T8

Intragroup analysis showed increased IgA levels at T8 (vs T4 *p* = 0.04) in the GLN group. On the contrary, the IgA level trend was always constant in the control group (*p* = 1). Intergroup analysis showed that *IgA* levels increased significantly in GLN vs control at *T0* (253.6±126.9 mg/mL vs 161±44.4 mg/mL; *p* = 0.03), at *T4* (256.4±128.7 mg/mL vs 163.2±43.5 mg/mL; *p* = 0.03) and at *T8* (280±148.3 mg/mL vs 153.6±32 mg/mL; *p* < 0.01) (Fig. 1).

**Fig. 2:**
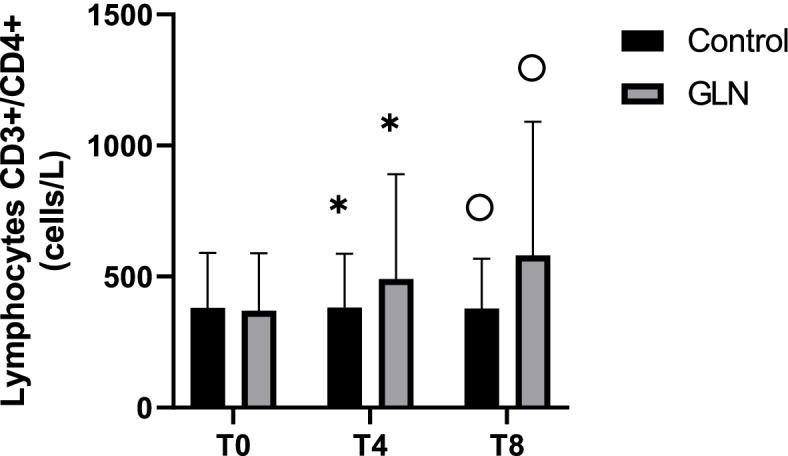
CD3+/CD4+ T helper lymphocyte levels in control and glutamine (GLN) groups. Data are presented as mean ± SD. *, °, and ^ indicate a significant difference in GLN vs control at T4 (*p* < 0.01) and T8 (*p* < 0.001); ˅, ”, and • indicate statistically highly significant (*p* < 0.001) in the GLN group

Intragroup analysis showed that in the GLN group CD3+/CD4+ T helper lymphocytes significantly increased in each time during the study period (*p* < 0.001), while in the control group CD3+/CD4+ T helper lymphocytes were stable over the time. Instead, intergroup analysis showed that CD3+/CD4+ T helper lymphocytes were 518.3±115.7 cells/μL in the GLN group and 519.6 ±162.8 cells/μL in control at baseline (*p* = 0.98) and their levels significantly increased in GLN vs the control group at T4 (767.6±232 cells/μL vs 539.7±165.5 cells/μL; *p* < 0.01) and T8 (903.2±257.2 cells/μL vs 555.6±157.7 cells/μL; *p* < 0.001) (Fig. 2).

**Fig. 3:**
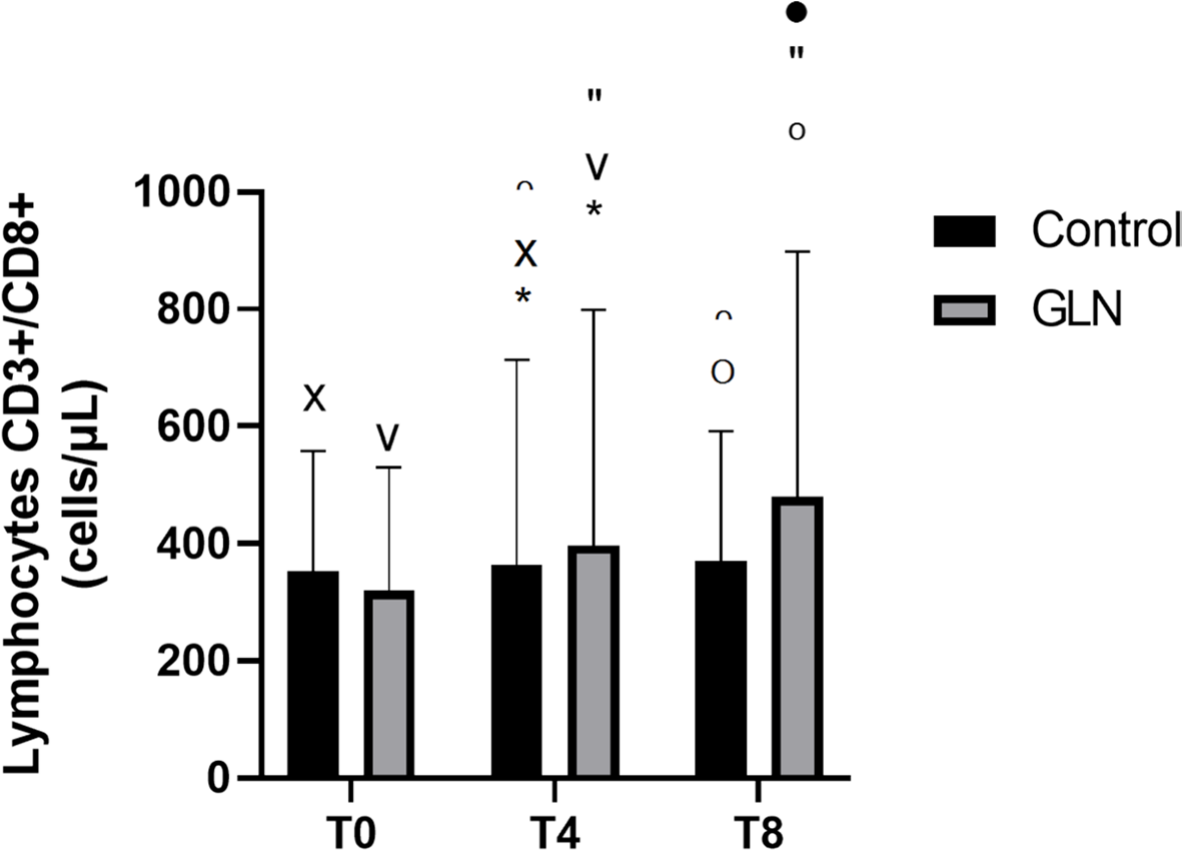
CD3+/CD8+ T suppressor lymphocyte levels in control and glutamine (GLN) groups. Data are presented as mean ± SD. * and °indicate a significant difference in GLN vs control at T4 (*p* < 0.05) and at T8 (*p* < 0.001); ˅, ”, and • indicate statistically significant (*p* < 0.001) in the GLN group; x and ∩ indicate a significant difference in the control group between T0 and T4 (*p* < 0.001) and T4 and T8 (*p* = 0.002)

Intragroup analysis showed that in the GLN group CD3+/CD8+ T suppressor lymphocytes highly increased in each time during the study period (*p* < 0.001), while in the control group they increased only at T4 (vs T0: *p* < 0.001) and T8 (vs T4: *p* = 0.002). Intergroup analysis showed that at T0, CD3+/CD8+ T suppressor lymphocytes were 498.5±153.2 cells/μL in the GLN group and 482.5±242 cells/μL in control (*p* = 0.8) and their levels significantly increased in GLN vs the control group at T4 (680.3±186.1 cells/μL vs 530.6±226.9 cells/μL; *p* < 0.05) and T8 (839.5±162.6 cells/μL vs 571.3±225.7 cells/μL; *p* < 0.001) (Fig. 3).

**Fig. 4:**
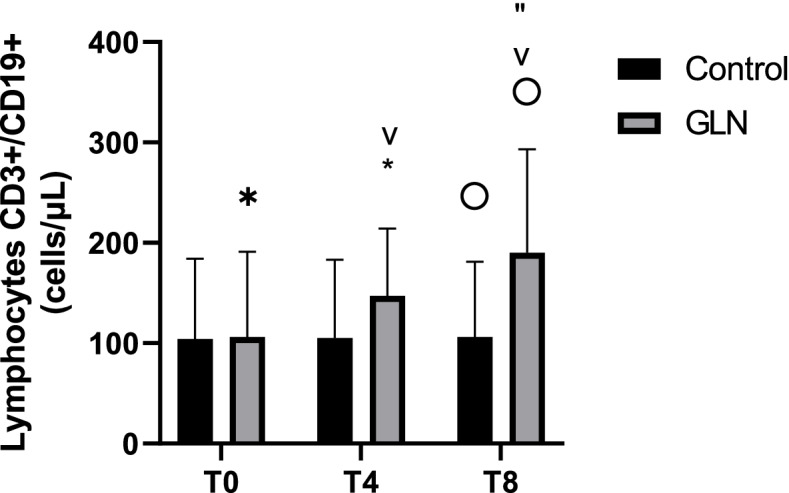
B lymphocyte CD3+/CD19+ levels in control and glutamine (GLN) groups. Data are presented as mean ± SD. "indicate a significant difference in GLN vs control at T8 (*p* = 0.01); *, ˅, and ” indicate statistically highly significant (*p* < 0.001) in the GLN group

Intragroup analysis showed that in the GLN group CD3+/CD19+ B lymphocytes significantly increased in each time during the study period (*p*< 0.001), while in the control group they were stable. Intergroup analysis showed at T0, CD3+/CD19+ B lymphocyte levels were 169.3±69 cells/μL in the GLN group and 157.6±65 cells/μL in control (*p* = 0.6) and increased significantly in GLN vs the control group only at *T8* (266.3±131.7 cells/μL vs 163.8±67.3 cells/μL; *p* = 0.01) (Fig. 4).

**Fig. 5:**
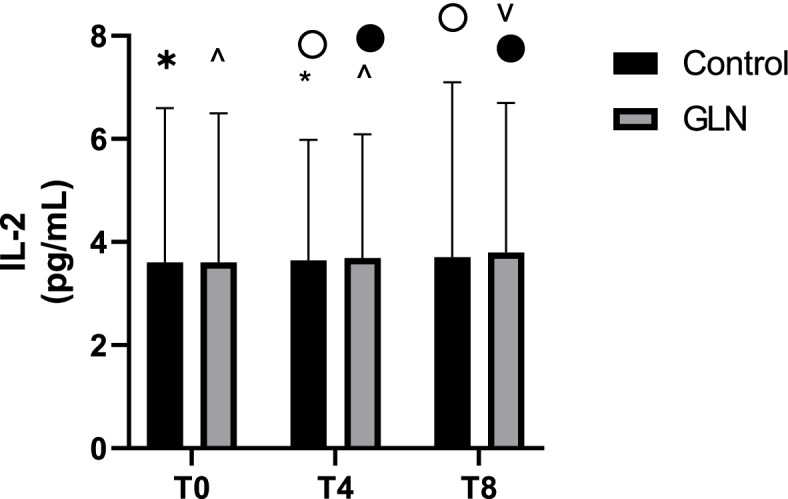
IL-2 levels in control and glutamine (GLN) groups. Data are presented as mean ± SD. ^, •, and ˅ indicate a significant difference in the GLN group (*p* < 0.05); * and ° indicate a significant difference in the control group between T0 and T4 (*p* = 0.002) and T4 and T8 (*p* = 0.002)

Intragroup analysis showed that in the GLN group IL-2 levels significantly increased in each time during the study period (*p* < 0.05), while in the control group they significantly increased only at T4 (vs T0: *p* = 0.002) and T8 (vs T4: *p* = 0.002) (Fig. 5). Instead, intergroup analysis showed that no differences were observed between GLN and control groups at *T0* (5.2±0.9 pg/mL vs 5.6±1.3pg/mL; *p* = 0.4), T4 (5.6±0.9pg/mL vs 5.9±1.4 pg/mL; *p* = 0.5) and *T8* (6. 2±0.9 pg/mL vs 6.1±1.5 pg/mL; *p* = 0.8).

**Fig. 6:**
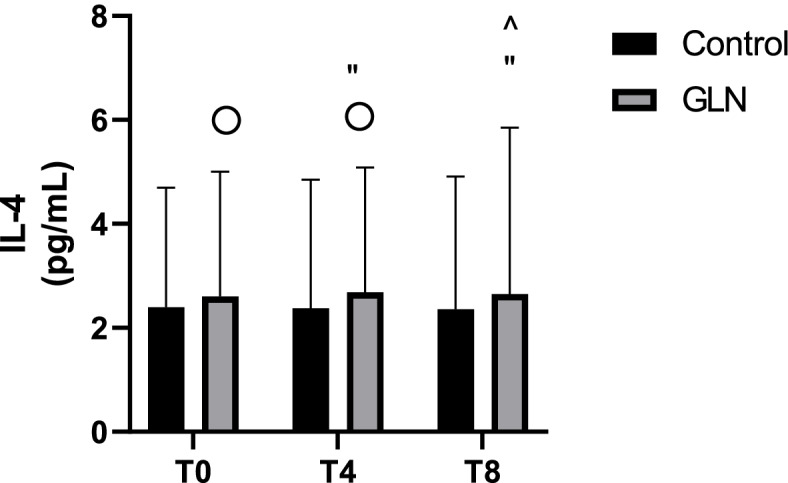
IL-4 levels in control and glutamine (GLN) groups. Data are presented as mean ± SD. °, ”, and ^ indicate a significant difference (*p* < 0.004) in the GLN group

Intragroup analysis showed higher IL-4 levels in each time point in the GLN group (*p*< 0.004) and constant IL-4 levels in the control group. In intergroup analysis, *IL-4* levels showed no significant differences when comparing GLN with the control group (Fig. 6).

## Discussion

Our study showed higher IgA levels (i), CD3+/CD4+ T helper lymphocytes and CD3+/CD8+ T suppressor lymphocytes (ii) in the GLN group vs the control group; IL-2 and IL-4 increased, but not significantly in the GLN group vs the control group (iii).

Our study is the first to investigate the IgA plasma level–mediated humoral immunity in polytrauma patients treated with GLN supplementation, showing higher IgA levels. IgA is the most abundant immunoglobulin in the human body and performs a very specialized role which involves mucosal immunity, development of tolerance and protection against infection. It is the key immunoglobulin in the respiratory and gastrointestinal tracts, which provide the first line of body defence [8].

Production of IgA is controlled by cytokine-producing T cells within the gut-associated lymphoid tissue (GALT) and possibly by cytokine released from the mucosa [9]. The two most important compartments in which IgA are located are blood and mucous secretions: in the blood, IgA is found as a monomer and is produced in the bone marrow by plasma cells that derive from B cells activated in the lymph nodes; in mucous tissues, IgA is secreted from IgA+ plasma cells after sensitization in the Peyer’s patches as a dimer, bound by a J chain, and associated with the polymeric immunoglobulin receptor. The resulting immunoglobulin is known as secretory IgA (sIgA) [10, 11]. It is well known that GLN affects intestinal production of sIgA in humans and many other mammals, such as mice, pigs, and rats, and has critical roles in intestinal homeostasis by regulating immune responses via multiple mechanisms [12–14].

Herein, we showed higher T and B lymphocyte levels in the GLN group. GLN appears to be fundamental in activation and proliferation of both CD3+/CD19+ B and CD3+/CD4+ and CD3+/CD8+ T lymphocytes. Specifically, it has been noticed that GLN enhances T lymphocytes’ functions, playing a crucial role in the Krebs cycle [15]. Additionally, recent data showed that T cell activation is associated with rapid GLN uptake, by an amino acid transporter, ASCT2, as well as in vitro and in vivo conditions [16].

Regarding the cytokine profile, we analysed IL-2 and IL-4, pro- and anti-inflammatory cytokines, respectively.

Concord to in vitro experiments, our results showed increased IL-2 and IL-4 levels in patients treated with GLN.

Chang et al. found that 0.6mM GLN significantly enhanced IL-2 and IL-4 levels in vitro. Furthermore, cytokine responses required the presence of optimal concentrations of GLN [11–17].

Another important item is the route of GLN administration (PN or EN). Although there are several reports of beneficial effects of GLN supplementation by the EN route in ICU patients [18, 19], there is always an uncertainty whether or not the patient has received the prescribed dose. In addition, the complete absorption of GLN in the upper part of the intestine leaves very little extra GLN to other tissues as almost nothing passes through the liver into the general circulation. So these are arguments that favour the GLN administration by the PN route in addition to EN [20]. For these reasons, we have chosen PN GLN supplementation in ICU polytrauma patients.

Regarding the timing and dose of GLN supplementation, our patients were treated by conventional EN enriched with 50mg/kg/IBW of intravenous GLN for 7 consecutive days, according to previous literature results. Recently, ESPEN guidelines recommend, in critically ill trauma, additional EN doses of GLN (0.2–0.3 g/kg/day) for the first 5 days with EN and in case of complicated wound healing for a longer period of 10 to 15 days [21].

This study showed a similar mortality and length of ICU stay in both polytrauma groups. However, contradictory findings have been recently reported in the literature regarding the linking between low or high GLN levels and mortality, so the debate is still open [22].

Our study showed that MV time was comparable between GLN and control groups.

Contrary, Ni et al. showed that in critical patients with acute liver injury MV time was shorter in the GLN group than in the control group but they could not claim that this result is strictly addicted to GLN supplement [23].

However, animal researches demonstrated that GLN preserved breath muscle strength and reduced tissue damage in the organs [24, 25]. Because of the retrospective nature of our study, we could not evaluate any ultrasonographic diaphragmatic measurement such as diaphragmatic inspiratory excursion, time to peak inspiratory amplitude of the diaphragm, diaphragmatic thickness (DT), DT difference, and diaphragm thickening fraction, during the study period [26].

Other limitations of our study were as follows: we could not measure GLN plasma levels, because of a lack of kits based on high-performance chromatography. It would be ideal to measure plasma glutamine levels of all patients prior to supplementation, but it is not routine practice in most clinical settings. Furthermore, we did not analyse infectious setting because of the high variability of antibiotic therapy.

## Conclusions

This study showed that there was an improvement in humoral and cell-mediated immunity with GLN supplementation in polytrauma ICU patients using recommended doses and it could be an important preliminary basis for a larger study.

The results of this study will provide pilot data for a larger clinical trial to investigate the exact mechanisms of any beneficial effects which have not been clearly understood.

Future research should confirm or refute whether a higher administration could translate any benefit for these patients.

Such studies should ensure that plasma GLN levels in treated patients are normalized.

## Data Availability

The database used and analysed during the current study is available from the corresponding author on reasonable request.

## Abbreviations

GLN: Glutamine
ICU: Intensive care unit
EN: Enteral nutrition
PN: Parenteral nutrition
IBW: Ideal body weight
SAPS II: Simplified Acute Physiology Score II
APACHES II: Acute Physiologic Assessment and Chronic Health Evaluation Score II
ISS: Injury Severity Score
SPSS: Statistical Package for the Social Sciences
MV: Mechanical ventilation
sIgA: Secretory immunoglobulin A
GALT: Gut-associated lymphoid tissue
DT: Diaphragmatic thickness
